# Mucin-1 Protein Is a Prognostic Marker for Pancreatic Ductal Adenocarcinoma: Results From the CONKO-001 Study

**DOI:** 10.3389/fonc.2021.670396

**Published:** 2021-07-27

**Authors:** Jana Käthe Striefler, Hanno Riess, Philipp Lohneis, Sven Bischoff, Annika Kurreck, Dominik Paul Modest, Marcus Bahra, Helmut Oettle, Marianne Sinn, Henrik Bläker, Carsten Denkert, Sebastian Stintzing, Bruno Valentin Sinn, Uwe Pelzer

**Affiliations:** ^1^Department of Hematology, Oncology, and Tumor Immunology, Charité–Universitätsmedizin Berlin, Corporate Member of Freie Universität Berlin, Humboldt-Universität zu Berlin, and Berlin Institute of Health, Berlin, Germany; ^2^Faculty of Medicine and University Hospital Cologne, Institute of Pathology, University of Cologne, Cologne, Germany; ^3^Department of Surgery, Charité –Universitätsmedizin Berlin, Corporate Member of Freie Universität Berlin, Humboldt-Universität zu Berlin, and Berlin Institute of Health, Berlin, Germany; ^4^Dayclinic for Oncology and Hematology, Outpatient Department of Medical Oncology, Friedrichshafen, Germany; ^5^University Medical Center Hamburg-Eppendorf, Oncology, Hematology and Bone Marrow Transplantation with the Section Pneumology (II Medical Clinic and Polyclinic), Hamburg, Germany; ^6^Institute of Pathology, Leipzig University Medicine, Leipzig, Germany; ^7^Institute of Pathology, University Clinic of Gießen and Marburg, Marburg, Germany; ^8^Institute of Pathology, Charité–Universitätsmedizin Berlin, Corporate Member of Freie Universität Berlin, Humboldt-Universität zu Berlin, and Berlin Institute of Health, Berlin, Germany

**Keywords:** pancreatic cancer, gemcitabine, MUC1, prognostic marker, CONKO 001 trial, adjuvant therapy

## Abstract

**Background:**

The Mucin-family protein, MUC1, impacts on carcinogenesis and tumor invasion. We evaluated the impact of MUC1 expression on outcome in a cohort of 158 patients with resected pancreatic ductal adenocarcinomas (PDAC) in the CONKO-001 study (adjuvant gemcitabine [gem] *vs*. observation [obs]).

**Methods:**

The percentage of MUC1-positive tumor cells by immunohistochemistry (IHC) and the staining intensity were evaluated by two observers blinded to outcome. The numeric values of both parameters were multiplied, resulting in an immunoreactivity score (IRS) ranging from 0 to 12. The level of MUC1 expression was defined as follows: IRS 0–4 (low) *vs* IRS >4 (high). Outcomes in terms of disease-free (DFS) and overall survival (OS) were evaluated by Kaplan–Meier method, log-rank tests and Cox regressions.

**Results:**

In total, tumors of 158 study patients were eligible for immunohistochemistry of MUC1. High cytoplasmic MUC1 expression was associated with impaired DFS and OS in the overall study population (hazard ratio (HR) for DFS: 0.49, 95% CI 0.31 to 0.78, p = .003; HR for OS: 0.46, 95% CI 0.29 to 0.73, p = .001). In the study arms, prognostic effects of MUC1 were also evident in the observation group (HR for DFS: 0.55; 95% CI 0.29 to 1.04, p = .062; HR for OS: 0.34, 95% CI 0.17 to 0.67, p = .001) and trending in the gem group (HR for DFS: 0.48, 95% CI 0.24 to 0.95, p = .041; HR for OS: 0.56, 95% CI 0.28 to1.11, p = .093).

**Conclusion:**

Our data suggest that MUC1 expression is a powerful prognostic marker in patients with PDAC after curatively intended resection.

## Highlights

- MUC1 is widely used as tumor marker especially in breast, ovarian, lung and pancreatic cancer.
- Low MUC1 expression is significantly associated with favorable prognosis in patients with pancreatic cancer after curatively intended resection.
- Prognostic impact of MUC1 is irrespective of active treatment *vs*. observation in the setting of the CONKO 001 trial.
- MUC1 expression might help to guide adjuvant treatment strategies and improve the outcome of patients at high risk of relapse and death.

## Introduction

### Pancreatic Cancer

Adjuvant chemotherapy is standard of care (SOC) in patients with PDAC after R0/R1 resection of the primary tumor with curative intent. Several regimens have been developed, of those gemcitabine monotherapy remains the standard for patients that are unfit for intensive combinations treatment (1–4).

### Role of Mucin-1

The transmembrane mucin glycoprotein Mucin-1 (MUC1), also known as CA 15-3, is a member of the mucin family of proteins expressed at the apical surface of epithelial cells. In cancer cells MUC1 accumulates within the mitochondria and the nucleus. The cytoplasmic tail of MUC1 serves as an adaptor protein connecting kinases and other cell signaling proteins, leading to increased cell proliferation, changes in adhesive state of the cell, invasion into the extracellular matrix and deregulation of apoptosis. MUC1 positive carcinomas are associated with a hyperactivation of critical signaling pathways such as mitogen-activated protein kinase (MAPK), phosphatidylinositol 3-kinase (PI3K/Akt) and wingless type (Wnt) pathway (5).

MUC1 as CA 15-3 is widely used as tumor marker especially in breast, ovarian, lung and pancreatic cancer. In breast cancer, MUC1 was shown to provide predictive information for therapy response and also for survival (6). Previous investigations of human tissue specimens suggested a crucial prognostic role for MUC1 in pancreatic adenocarcinoma (7).

Furthermore, as recently shown in murine pancreatic cancer cell lines MUC1 is a potential therapeutic target (8) with small molecules in early clinical development, stimulating the characterization of a potential target population in pancreatic cancer (9).

To the best of our knowledge, no data from prospective clinical trials evaluating the expression and prognostic role of MUC1 in pancreatic cancer patients are available, yet. The aim of our analysis was to evaluate the impact on outcome of MUC1 in the CONKO-001 trial allowing for assessment of effects with and without adjuvant therapy.

### Study Population

CONKO-001 was a phase III trial, where 368 patients with pancreatic adenocarcinoma were randomized to an adjuvant treatment with gemcitabine or to observation only after a curatively intended resection.

We aimed to demonstrate that low MUC1 expression is a valuable prognostic factor in pancreatic cancer patients. As this analysis is unplanned and exploratory, the results should be interpreted as such.

## Methods

The Reporting Recommendations for Tumor Marker Prognostic Studies (REMARK) criteria were followed for reporting this study.

### Study

Baseline data of CONKO-001: The prospective randomized phase III CONKO-001 trial investigated the role of an adjuvant treatment with gemcitabine as compared to observation. A total of 368 patients with completely resected pancreatic cancer (R0 and R1 resection) were recruited between July 1998 and December 2004. Gemcitabine (1,000 mg/m²) was given for 6 months in an outpatient setting. Follow-ups were scheduled in eight weekly intervals. Please refer to the existing primary publications of the trial for details (10, 11). The study was approved by the institutional review committee (trial registration isrctn.org Identifier: *ISRCTN34802808*).

### Patients

In total, 354 out of 368 patients were included into the survival analysis (gem: n = 179, obs: n = 175). Archival tumor tissue was available from 165 cases.

### MUC1

**Figure 1:**
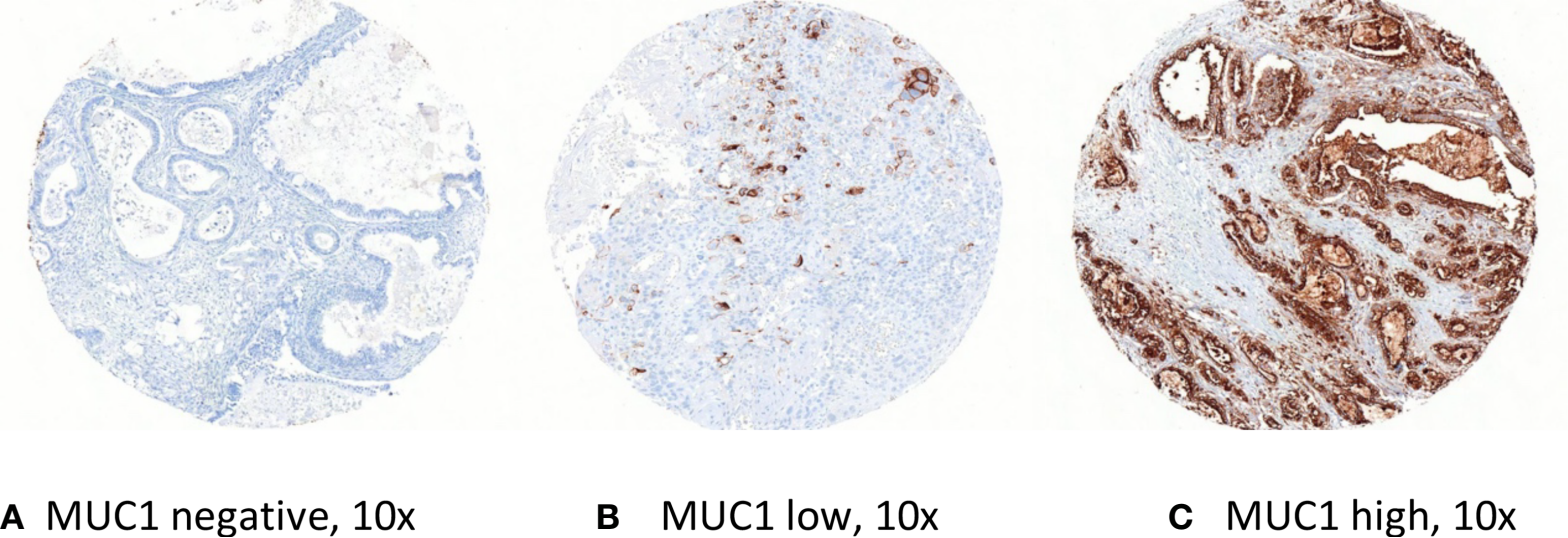
Representative TMAs **(A)** MUC1 negative, **(B)** MUC1 low, and **(C)** MUC1 high.

Immunohistochemical staining for Mucin-1 was carried out on tissue microarrays (TMAs) according to standard procedures (1:200; clone MA695; Leica Biosystems Newcastle, Ltd, Newcastle Upon Tyne, UK). To reduce effects of intratumoral heterogeneity, three representative 1-mm-tissue cores (0.785 mm^2^) were selected for the construction of tissue microarrays using a manual tissue microarrayer (Beecher Instruments, Sun Prairie, Wisconsin, USA). The stained slides were digitalized (Mirax Scan, Zeiss, Jena, Germany) and evaluated by virtual microscopy using the VMScope Silde Explorer (VMScope, Berlin, Germany) by two observers who were blinded to clinical outcome (MS, BVS). The percentage of positive tumor cells (0% = 0, 1–10% = 1, 11–50% = 2, 51–80% = 3, 81–100% = 4) and the staining intensity (negative = 0, weak = 1, moderate = 2, strong = 3) were evaluated [see **Figure 1**: *Representative TMAs (* ***A*** *) MUC1 negative, (* ***B*** *) MUC1 low, and (* ***C*** *) MUC1 high*]. For quantification of the expression level we used the well-established immunoreactivity score (IRS) ranging from 0 to 12, who is calculated by multiplication of the numeric values of both parameters.

### Statistical Considerations

For exploratory statistical analysis, two groups with low or high MUC1 expression were defined based on data distribution (IRS 0–4 *vs*. IRS >4). For determination of the cut-off separating most precisely survival differences an publicly accessible online tool was used (https://molpathoheidelberg.shinyapps.io/CutoffFinder_v1/), for more detailed information please refer to (12). Kaplan–Meier analyses for disease-free survival (DFS) and overall survival (OS) were performed according to MUC1 expression. In multivariable Cox regressions standard clinical and biomarker characteristics (age, sex, treatment arm, T stage, nodal status, grading, resection margin, and Karnofsky index) were investigated.

Disease-free survival (DFS) was defined as time from study entry to local or distant disease relapse, overall survival (OS) as time from study entry to death of any cause. The relation of MUC1 expression with clinical and pathological tumour characteristics was evaluated using χ^2^-tests. The Kaplan–Meier method with log-rank tests was used for univariable survival analyses. Cox regressions were used for multivariable survival models. In general, P-values <0.05 (calculated 2-sided) were considered significant.

## Results

### MUC1 Analyzed Subpopulation

**Figure 2:**
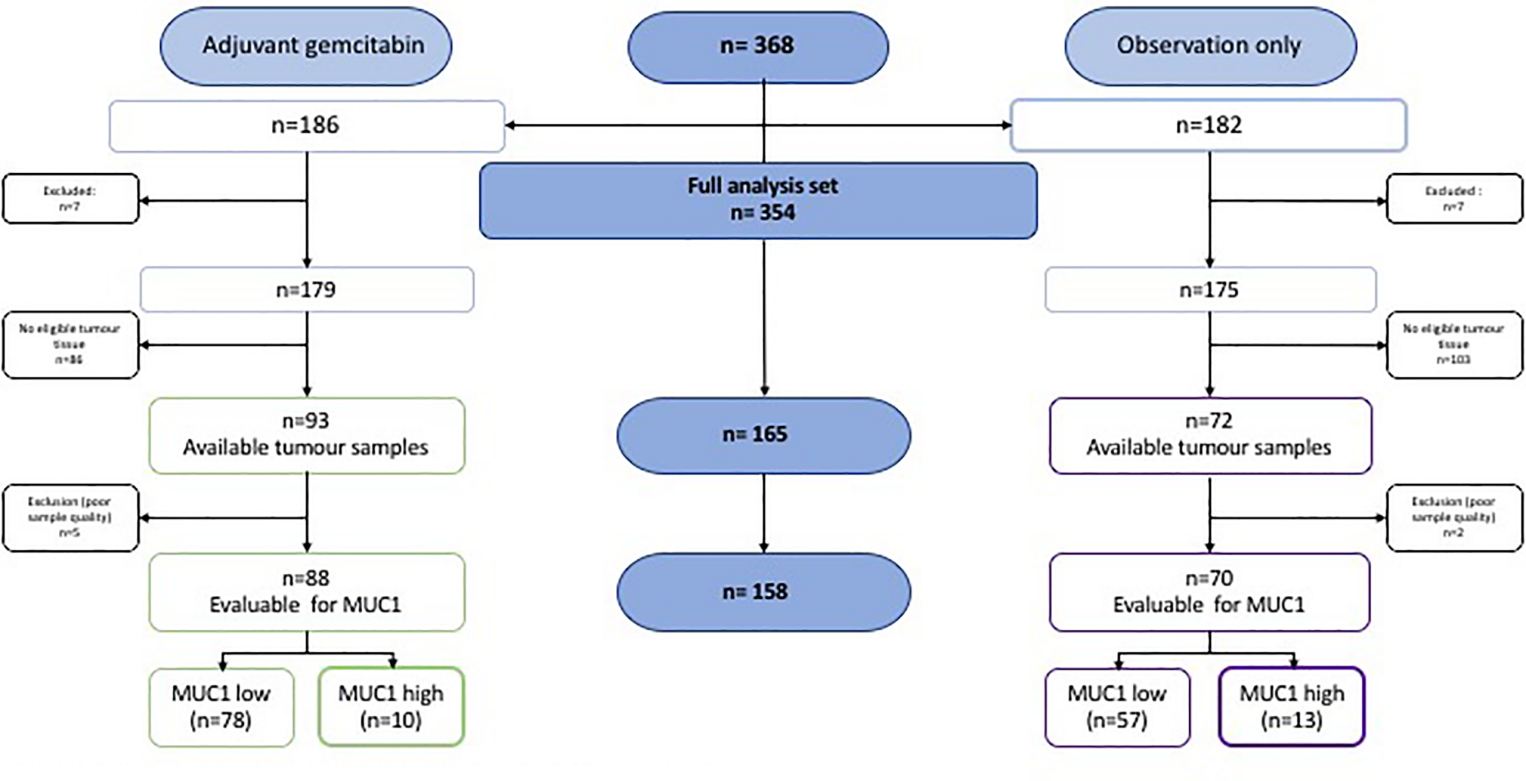
CONSORT diagram MUC1 in CONKO 001.

In total, 368 patients were enrolled in the CONKO 001 trial. N = 186 were randomized to the gemcitabine (gem) group and n = 182 to the observation (obs) group. Of those, in n = 165 cases tumor tissue was available for analysis of MUC1 expression. Of these 165 cases, seven samples (gem n = 5, obs n = 2) were excluded from the analysis due to poor quality (see **Figure 2**: *CONSORT diagram MUC1 in CONKO 001*), resulting in 88 cases of the gemcitabine-group and 70 cases of the observational group in the analyzed population. The patients´ and the tumor characteristics were well balanced across both groups and did not differ from the overall CONKO 001 study population. Please refer to **Table 1**: *Baseline patients and tumor characteristics*.

**Table 1:** Shown are the relevant clinical and histopathological features of the studied subgroups in which MUC1 expression level was analyzed.

Clinical and histopathological features	Overall study population (n = 354)			MUC1 analyzed subpopulation (n = 158)	
	Overall	Gemcitabine n = 179	Observation n = 175	Gemcitabine n = 88	Observation n = 70
**Age**					
median (range), y	62 (34–82)	62 (34–82)	62 (36–81)	63 (37–80)	60 (36–81)
<65 years	219 (62)	115 (61)	104 (59)	51	48
≥65 years	135 (38)	64 (36)	71 (41)	37	22
**Karnofsky performance status scale**					
median (range), %	80 (50–100)	80 (60–100)	80 (50–100)	90 (60–100)	80 (50–100)
>80%	230 (65)	129 (72)	101 (58)	63 (72)	46 (66)
≤80%	124 (35)	50 (28)	74 (42)	25 (28)	24 (34)
**Gender, (%)**					
male	203 (57)	105 (59)	98 (56)	54 (61)	40 (57)
female	151 (43)	74 (41)	77 (44)	34 (39)	30 (43)
**T stage, (%)**					
T1–2	49 (14)	25 (14)	24 (14)	9 (10)	7 (10)
T3–4	305 (86)	154 (86)	151 (86)	79 (90)	63 (90)
**Nodal status, (%)**		5	4		
N−	100 (28)	2 (29)	8 (27)	18 (20)	16 (23)
N+	254 (72)	127 (71)	127 (73)	70 (80)	54 (77)
**Grading, (%)**					
G1–2	218 (63)	113 (64)	105 (61)	53 (61)	37 (54)
G3	130 (37)	63 (36)	67 (39)	34 (39)	32 (46)
**Resection margin, (%)**					
R0	293 (83)	145 (81)	148 (85)	73 (83)	55 (79)
R1	61 (17)	34 (19)	27 (15)	15 (17)	15 (21)

Grading was not available in all cases. T1–2, T1–2 stage; T3–4; T3–4 stage; N−, nodal negative stage; N+ nodal positive stage; R0, R0 resection stage; R1, R1 resection stage.

### Archival Tissue Samples and Staining Results

As described above, sufficient quality of immunohistochemical staining of MUC1 expression was achieved in 158 of 165 tumor samples (gem: n = 88, obs: n = 70). In the gem arm, n = 78 samples were evaluated as low, and n = 10 as high MUC1 expression, respectively. In those patients randomized to observation only, n = 57 were assessed as low, and n = 13 as high MUC1 expression. Cytoplasmic staining was the most frequently observed pattern (please refer to **Figure 2**: *CONSORT diagram MUC1 in CONKO 001*).

### Clinical and Histopathological Characteristics of the MUC1 Subpopulation

Patients with low *vs*. high MUC1 expression were found in similar frequencies in both arms of the trial (MUC1 low: gem n = 78, 58%, obs n = 57, 42% *vs*. MUC1 high: gem n = 10, 43% obs n = 13, 57%). The only baseline characteristic that appeared to be associated with MUC1 expression was age. The frequency of patients under the age of 65 was clearly higher in the MUC1 low group (MUC1 low: n = 90, 67% *vs*. MUC1 high: n = 9, 39%; *p* = .018). No relevant differences in other clinical and histopathological features were found in the MUC1 low *vs*. high cohort (please refer to **Table 2**: *Association of MUC1 expression level and selected patient characteristics*).

**Table 2:** Association of MUC1 expression level and selected patient characteristics.

MUC1 analyzed subpopulation, n = (%)			
Clinical and histopathological features	MUC1 low n = 135 (85)	MUC1 high n = 23 (15)	p =
**Age**			.018
median (range), y	61 (36–79)	67 (37–81)	
<65 years	90 (67)	9 (39)	
≥65 years	45 (33)	14 (61)	
**Karnofsky performance status scale**			.999
median (range), %	80 (50–100)	80 (70–100)	
>80%	93 (69)	16 (70)	
≤80%	42 (31)	7 (30)	
**Gender, (%)**			.649
male	79 (59)	15 (65)	
female	56 (41)	8 (35)	
**T stage, (%)**			.471
T1–2	15 (11)	1 (4)	
T3–4	120 (89)	22 (96)	
**Nodal status, (%)**			.586
N−	28 (21)	6 (26)	
N+	107 (79)	17 (74)	
**Grading, (%)**			.171
G1–2	80 (60)	10 (43)	
G3	53 (40)	13 (57)	
**Resection margin, (%)**			.152
R0	112 (83)	16 (70)	
R1	23 (17)	7 (30)	
**Treatment arm, (%)**			.257
Gemcitabin	78 (58)	10 (43)	
Observation	57 (42)	13 (57)	

Grading was not available in all cases. T1–2, T1–2 stage; T3–4; T3–4 stage; N−, nodal negative stage; N+ nodal positive stage; R0, R0 resection stage; R1, R1 resection stage.

### Survival

**Figure 3:**
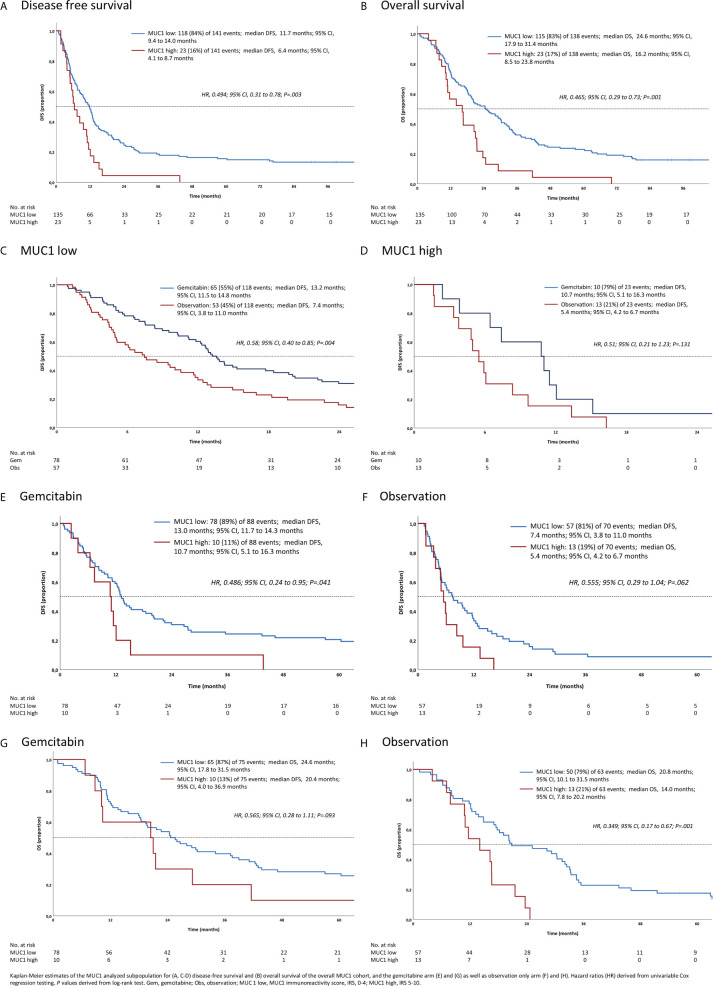
Survival analyses in subgroups for **(A–D)** MUC1 low *vs*. high, and gemcitabine *vs*. observation **(E–H)**.

In the overall study population, low cytoplasmic MUC1 expression was associated with favorable DFS and OS (hazard ratio (HR) for DFS: 0.5, 95% CI 0.31 to 0.78, p = .003; HR for OS: 0.46, 95% CI 0.29 to 0.74, p = .001) see **Figures 3A, B**: *Survival analyses in subgroups for MUC1 low vs. high*.

Referring to the MUC1 low subpopulation, disease free survival was significantly improved in patients treated with gemcitabine compared to those of the observation only group (HR for DFS: 0.58; 95% CI 0.40 to 0.85, p = .004). By contrast, there was no relevant difference in overall survival observed (HR for OS: 0.51, 95% CI 0.21 to 1.23, p = .131), see **Figures 3C, D**: *Survival analyses in subgroups for MUC1 low vs. high*.

In the observation group, we found favorable prognostic effects of low MUC1 expression (HR for DFS: 0.55; 95% CI 0.29 to 1.04, p = .062; HR for OS: 0.35, 95% CI 0.18 to 0.68, p = .001) as well as a strong trend for improved survival in the gemcitabine group (HR for DFS: 0.48; 95% CI 0.24 to 0.96, p = .041; HR for OS: 0.56, 95% CI 0.28 to 1.11, p = .093), see **Figures 3E–H**: *Survival analyses in subgroups for gemcitabine vs. observation.*

### Multivariable Analysis

**Figure 4:**
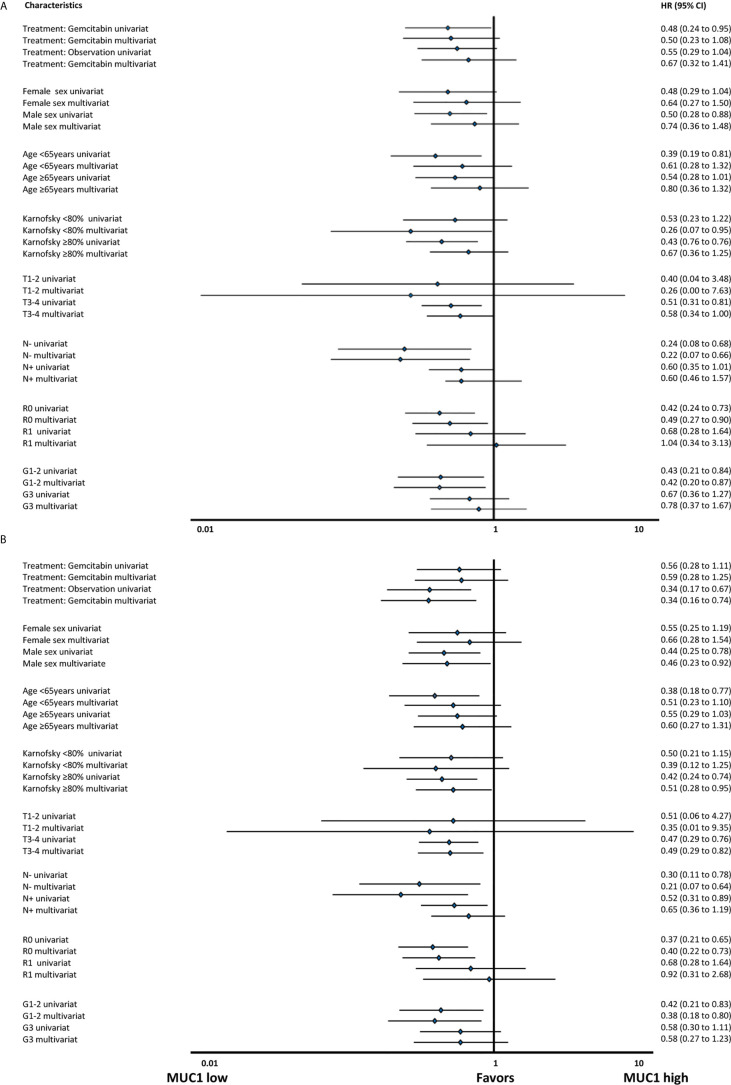
Survival analyses in subgroups for **(A)** disease free survival and **(B)** overall survival.

In multivariable Cox regressions including standard clinical and biomarker characteristics, only treatment arm was independently predictive for DFS (HR 0.49 [95% CI: 0.29–0.83]; *p* =  .008), whereas MUC1 (HR 0.47 [95% CI: 0.22–0.99]; *p* =  .05) and grading (HR 0.61 [95% CI: 0.37–1.00]; *p* =  .05) were strongly trending to predict OS, respectively, see also **Figure 4** for detailed exploratory analyses: *Survival analyses in subgroups for (* ***A*** *,* ***B*** *) disease free survival and (* ***C*** *,* ***D*** *) overall survival*.

## Discussion

In the presented analysis we explored the prognostic impact of MUC1 expression in pancreatic cancer patients in the context of a controlled randomized trial with a highly characterized population with mature outcome data. This cohort allows for rather valid data generation also acknowledging the retrospective and hypothesis-generating character of the article.

In the CONKO 001 trial, low MUC1 expression level appeared more frequent in patients under the age of 65 years. The reason thereof is not fully understood although this observation corresponds to pre-existing data (6, 13).

The remaining clinical and histopathological features did not significantly differ between the MUC1 low and MUC1 high cohort, respectively. Concurrently, in other solid cancer types MUC1 expression level does not correlate with specific clinicopathological parameters. As an exception, in several studies of breast cancer, MUC1 positivity was found to correlate with adverse metastases stages, nodal status and histological grading as well as hormone insensitivity (12–14). By contrast, the expression level of MUC1 in colon cancer and gastric cancer apparently did not correlate with any clinicopathological parameter but still is an independent marker of prognosis (15, 16).

Accordingly, low MUC1 expression levels were clearly associated with favorable outcome of patients. The positive prognostic effect was slightly higher in patients treated with gemcitabine in regard to DFS only. In contrast, significance could not be demonstrated for all subsets, likely due to limited sample size. Thus, this finding appeared evident irrespective of study arm and endpoint (DFS, OS) in the overall study population. Importantly, exploratory subgroup analyses did not identify specific patients in which the prognostic effect was more or less pronounced, indicating that MUC1 could be a relatively general biomarker of prognosis.

No relevant interaction of treatment efficacy (with gemcitabine) and MUC1 expression for OS was observed, suggesting that MUC1 is not predictive of gemcitabine efficacy. Interestingly, this is somehow contrasted by (preclinical) reports that suggest an association of gemcitabine efficacy with high MUC1 expression. An association of MUC1 upregulation and gemcitabine resistance in pancreatic tumor cells was described in several preclinical investigations (5, 17, 18).

However, gemcitabine monotherapy is no longer the undisputed standard of care in the adjuvant setting of PDAC for patients with a sufficient performance status for a combination therapy. Therefore, the impact of MUC1 expression level might be different if intensified cytostatic regimes are administered. However, our data are able to confirm the pure prognostic effect of MUC1 expression due to the comparison of adjuvant chemotherapy to observation. Certainly, the prognostic role of MUC1 in the context of resectable pancreatic cancer needs to be validated by other study groups. For instance, it is unclear to which extent our findings can be generalized to cohorts using more intensive adjuvant regimens such as mFOLFIRINOX, and gemcitabine plus capecitabine (1, 2).

Furthermore, it might be concluded that the poor outcome of patients with high MUC1 expression could be improved with the mentioned more active treatment regimens. Potentially, MUC1 high expressing PDAC defines a high-risk subgroup in the adjuvant setting. Therefore, intensive treatment approaches with active surveillance should be evaluated prospectively in this subgroup.

Additionally, the specific localization and expression level of MUC1 in PDAC differing from healthy pancreatic tissue, enables multiple immunotherapeutic strategies. Interestingly, several antibodies targeting MUC1 are currently in development (19), as well as vaccine formulations that may increase mucin-specific cytotoxic T-lymphocytes (20). Finally, also in the context of CAR T cells, MUC1-specific T-cells (TAB004) are already tested in a phase I clinical trial in patients with advanced solid tumors [NCT04137900].

Several limitations of this analysis should be considered in the interpretation of the data: The biomaterial was collected not before completion of the trial. Thus, of the initial 354 patients included in the CONKO 001 survival analysis, there was archival tumor tissue in only n = 165 cases available. The number was further reduced due to poor quality to n = 158 (45%) samples of whom the tissue microarray were constructed. However, the clinical and histopathological features of the here presented subset are comparable to the overall intention to treat population. A further limitation might be inherent in the tissue microarray approach which is limited in its ability to assess tissue and tumour heterogeneity. Therefore, we examined tissue microarray cores in triplicate to overcome sample bias. Due to the shortage of tissue samples, comparison of MUC1 with various other potential biomarkers relating to their respective prognostic role was not realizable. Referring to the classification of MUC1 expression level by the IRS, there exists no well-established standard. Thus, the here presented cut off might serve as a reference for subsequent analyses in resectable pancreatic cancer. For this analysis, we assumed a good correlation of the immunohistochemical staining with the MUC1 expression on the transcriptional level which as it was shown for other solid cancer types e.g. breast cancer (6). The limited quantity of available biomaterial made it impossible to analyze the respective gene expression in our cohort and thus represents a potential bias of our study. Comparison with transcriptomic signatures might further clarify the prognostic value of MUC1 in pancreatic cancer (21–25). However, to the best of our knowledge, there exists no such gene expression score for resectable pancreatic cancer yet. In addition, none of the previously published transcriptomic signatures is sufficient as sole basis for therapeutic decision making. Thus, an evaluation of gene expression patterns is urgently needed to be implemented prospectively into adjuvant trials. Correlation of our data, and MUC1 expression level respectively, with transcriptomic signatures might contribute to the development of reproducible prognostic scores. Naturally, stratification of study arms and MUC1 expression heavily limits the sample size in subgroups, resulting in small numbers that may generate hypothesis but do not allow definite conclusions.

## Conclusion

Low MUC1 expression is significantly associated with favorable DFS and OS in patients with pancreatic cancer after curatively intended resection. This finding appeared to be irrespective of active treatment *vs*. observation in the setting of the CONKO 001 trial. No conclusions of a potential predictive value can be drawn. Future studies should clarify if the negative prognostic impact of high MUC1 expression can be generalized and to which extent more intensive adjuvant treatment strategies such as the widely used mFOLFIRINOX improve the outcome of patients at high risk of relapse and death.

## Data Availability Statement

The raw data supporting the conclusions of this article will be made available by the authors, without undue reservation.

## Author Contributions

JS had full access to all of the data in the study and takes responsibility for the integrity of the data and the accuracy of the data analysis. Concept and design: JS, MS, BS, and UP. Acquisition, analysis or interpretation of data: JS, UP, and BS. Drafting of the manuscript: JS. Critical revision of the manuscript for important intellectual content: BS, DM, and UP. Statistical analysis: JS, SB, DM, and UP. Administrative, technical, or material support: JS, HO, MB, HB, CD, SS, UP, and BS. All authors contributed to the article and approved the submitted version.

## Conflict of Interest

The authors declare that the research was conducted in the absence of any commercial or financial relationships that could be construed as a potential conflict of interest.

## Abbreviations

CONSORT, Consolidated Standards of Reporting Trials; DFS, disease-free survival; e.g., exempli gratia; gem, gemcitabine; HR, hazard ratio; IRS, immunoreactivity score; MAPK, mitogen-activated protein kinase; mFOLFIRINOX, modified FOLFIRINOX regime (fluorouracil, leucovorin, irinotecan, oxaliplatin); MUC1, Mucin-1; OS, overall survival; obs, observation; PDAC, pancreatic ductal adenocarcinoma; PI3K/Akt, phosphatidylinositol 3-kinase; REMARK, Reporting Recommendations for Tumor Marker Prognostic Studies; SOC, standard of care; TMA, tissue microarray; Wnt, wingless type; 95%CI, 95% confidence interval.
