# Supplementary material for: Mucin-1 Protein Is a Prognostic Marker for Pancreatic Ductal Adenocarcinoma: Results From the CONKO-001 Study
Source: Front Oncol. 2021 Jul 27;11:670396. doi: 10.3389/fonc.2021.670396 (PMC8354141; doi:10.3389/fonc.2021.670396)
Supplement: Supplementary file 1 [file DataSheet_1.pdf]

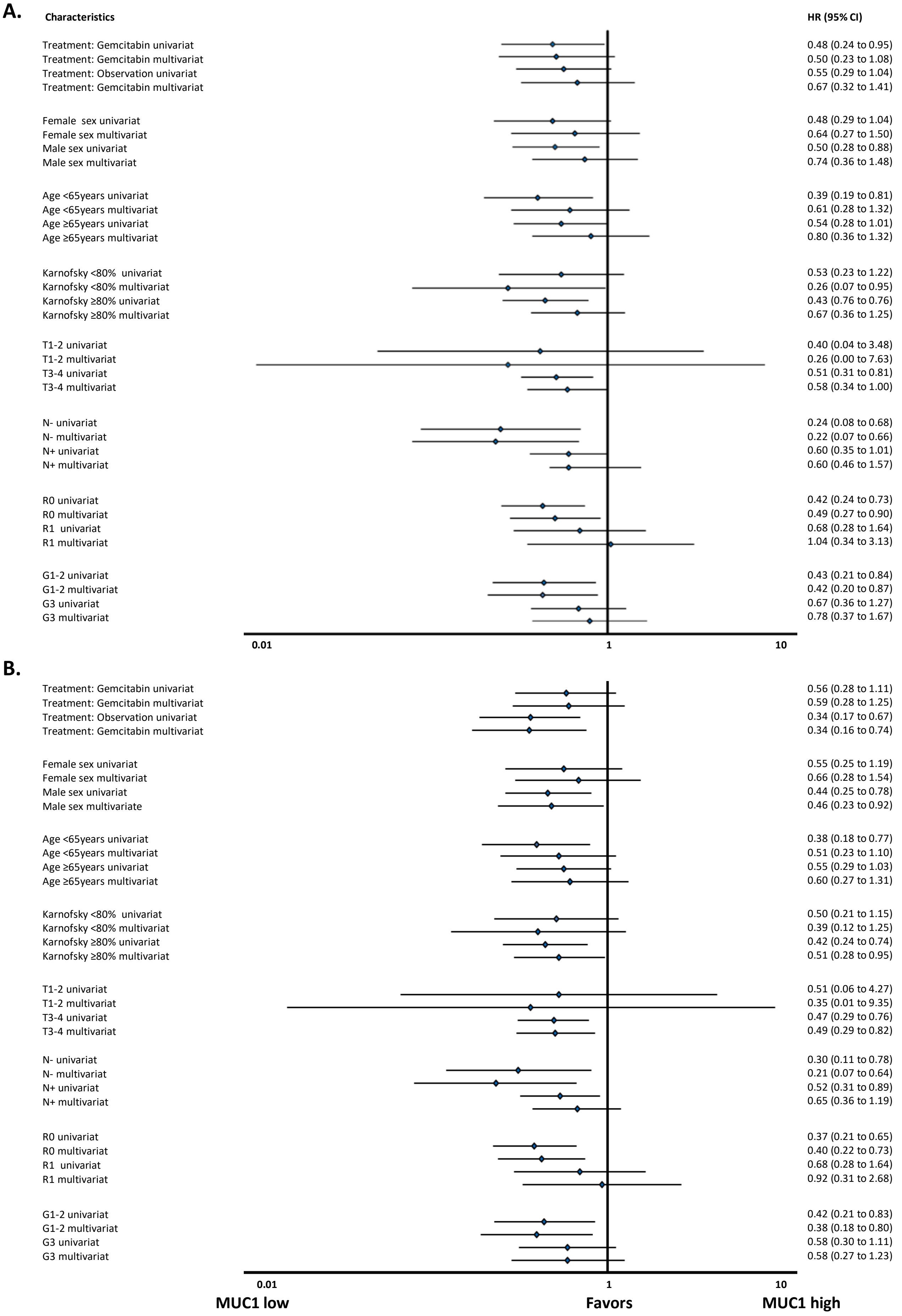
A.
Characteristics
HR (95% CI)
Treatment: Gemcitabin univariat
0.48 (0.24 to 0.95)
Treatment: Gemcitabin multivariat
0.50 (0.23 to 1.08)
Treatment: Observation univariat
0.55 (0.29 to 1.04)
Treatment: Gemcitabin multivariat
0.67 (0.32 to 1.41)
Female sex univariat
0.48 (0.29 to 1.04)
Female sex multivariat
0.64 (0.27 to 1.50)
Male sex univariat
0.50 (0.28 to 0.88)
Male sex multivariat
0.74 (0.36 to 1.48)
Age <65years univariat
0.39 (0.19 to 0.81)
Age <65years multivariat
0.61 (0.28 to 1.32)
Age ≥65years univariat
0.54 (0.28 to 1.01)
Age ≥65years multivariat
0.80 (0.36 to 1.32)
Karnofsky <80% univariat
0.53 (0.23 to 1.22)
Karnofsky <80% multivariat
0.26 (0.07 to 0.95)
Karnofsky ≥80% univariat
0.43 (0.76 to 0.76)
Karnofsky ≥80% multivariat
0.67 (0.36 to 1.25)
T1-2 univariat
0.40 (0.04 to 3.48)
T1-2 multivariat
0.26 (0.00 to 7.63)
T3-4 univariat
0.51 (0.31 to 0.81)
T3-4 multivariat
0.58 (0.34 to 1.00)
N- univariat
0.24 (0.08 to 0.68)
N- multivariat
0.22 (0.07 to 0.66)
N+ univariat
0.60 (0.35 to 1.01)
N+ multivariat
0.60 (0.46 to 1.57)
R0 univariat
0.42 (0.24 to 0.73)
R0 multivariat
0.49 (0.27 to 0.90)
R1 univariat
0.68 (0.28 to 1.64)
R1 multivariat
1.04 (0.34 to 3.13)
G1-2 univariat
0.43 (0.21 to 0.84)
G1-2 multivariat
0.42 (0.20 to 0.87)
G3 univariat
0.67 (0.36 to 1.27)
G3 multivariat
0.78 (0.37 to 1.67)
0.01
1
10
B.
Treatment: Gemcitabin univariat
0.56 (0.28 to 1.11)
Treatment: Gemcitabin multivariat
0.59 (0.28 to 1.25)
Treatment: Observation univariat
0.34 (0.17 to 0.67)
Treatment: Gemcitabin multivariat
0.34 (0.16 to 0.74)
Female sex univariat
0.55 (0.25 to 1.19)
Female sex multivariat
0.66 (0.28 to 1.54)
Male sex univariat
0.44 (0.25 to 0.78)
Male sex multivariate
0.46 (0.23 to 0.92)
Age <65years univariat
0.38 (0.18 to 0.77)
Age <65years multivariat
0.51 (0.23 to 1.10)
Age ≥65years univariat
0.55 (0.29 to 1.03)
Age ≥65years multivariat
0.60 (0.27 to 1.31)
Karnofsky <80% univariat
0.50 (0.21 to 1.15)
Karnofsky <80% multivariat
0.39 (0.12 to 1.25)
Karnofsky ≥80% univariat
0.42 (0.24 to 0.74)
Karnofsky ≥80% multivariat
0.51 (0.28 to 0.95)
T1-2 univariat
0.51 (0.06 to 4.27)
T1-2 multivariat
0.35 (0.01 to 9.35)
T3-4 univariat
0.47 (0.29 to 0.76)
T3-4 multivariat
0.49 (0.29 to 0.82)
N- univariat
0.30 (0.11 to 0.78)
N- multivariat
0.21 (0.07 to 0.64)
N+ univariat
0.52 (0.31 to 0.89)
N+ multivariat
0.65 (0.36 to 1.19)
R0 univariat
0.37 (0.21 to 0.65)
R0 multivariat
0.40 (0.22 to 0.73)
R1 univariat
0.68 (0.28 to 1.64)
R1 multivariat
0.92 (0.31 to 2.68)
G1-2 univariat
0.42 (0.21 to 0.83)
G1-2 multivariat
0.38 (0.18 to 0.80)
G3 univariat
0.58 (0.30 to 1.11)
G3 multivariat
0.58 (0.27 to 1.23)
0.01
1
10
MUC1 low
Favors
MUC1 high

Supplementary Figure 1: Survival analyses in subgroups for (A) disease free survival and (B) overall survival

Efficacy analyses in subgroups for (A) disease free survival and (B) overall survival. Forest plots with indicated analyses: (A) hazard ratios (HRs) for progression or death with 95% CIs, and (B) HRs for death with 95% CIs. Karnofsky, Karnofsky performance Status Scale; T1-2, T1-2 stage; T3-4; T3-4 stage; N-, nodal negative stage; N+ nodal positive stage; R0, R0 resection stage; R1, R1 resection stage.
